# (*anti*-Chlorido­thio­semicabazide-κ*S*)bis­(tri­phenyl­phosphane-κ*P*)copper(I) 0.48-hydrate

**DOI:** 10.1107/S1600536813008556

**Published:** 2013-04-05

**Authors:** Ruthairat Nimthong, Chaveng Pakawatchai, Nutchanat Phongphayak, Yupa Wattanakanjana

**Affiliations:** a Department of Chemistry and Center of Excellence for Innovation in Chemistry, Faculty of Science, Prince of Songkla University, Hat Yai, Songkhla 90112, Thailand; b Department of Chemistry, Faculty of Science, Prince of Songkla University, Hat Yai, Songkhla 90112, Thailand

## Abstract

In the mononuclear title complex, [CuCl(CH_5_N_3_S)(C_18_H_15_P)_2_]·0.48H_2_O, the Cu^I^ ion is in a slightly distorted tetra­hedral coordination geometry formed by two P atoms from two tri­phenyl­phosphane ligands, one S atom from a thio­semicarbazide ligand and one chloride anion. An intra­molecular N—H⋯N hydrogen bond [graph-set motif *S*(5)] stabilizes the thio­semicarbazide ligand in its *anti* conformation, and an intra­molecular N—H⋯Cl hydrogen bond between the hydrazine N—H group and the chloride anion influences the arrangement and orientation of the ligands around the metal center. A weak intra­molecular C—H⋯Cl hydrogen bond is also present. In the crystal, complex mol­ecules are connected through N—H⋯Cl hydrogen bonds originating from the amide –NH_2_ group, and through O—H⋯S and O—H⋯Cl hydrogen bonds involving the solvent water mol­ecule. Both the direct N—H⋯Cl hydrogen bonds as well as the bridging hydrogen bonds mediated by the water mol­ecule connect the complex mol­ecules into zigzag chains that propagate along [010]. The solvent water mol­ecule is partially occupied, with a refined occupancy of 0.479 (7).

## Related literature

For the coordination of thio­semicarbazide and thio­semicarbazones with metal complexes, see: Andreetti *et al.* (1970 ▶); Chattopadhyay *et al.* (1991 ▶); Jia *et al.* (2008*a* ▶,*b* ▶); Villa *et al.* (1972*a* ▶,*b* ▶); Qirong *et al.* (1987 ▶). For potential applications of related complexes, see: Alagarsamy & Parthiban (2011 ▶); Kowol *et al.* (2007 ▶); Pelosi (2010 ▶); Yu *et al.* (2009 ▶); Wattanakanjana *et al.* (2012 ▶). For hydrogen-bond graph-set motifs, see: Bernstein, *et al.* (1995 ▶). For a description of the Cambridge Structural Database (CSD), see: Allen (2002 ▶).
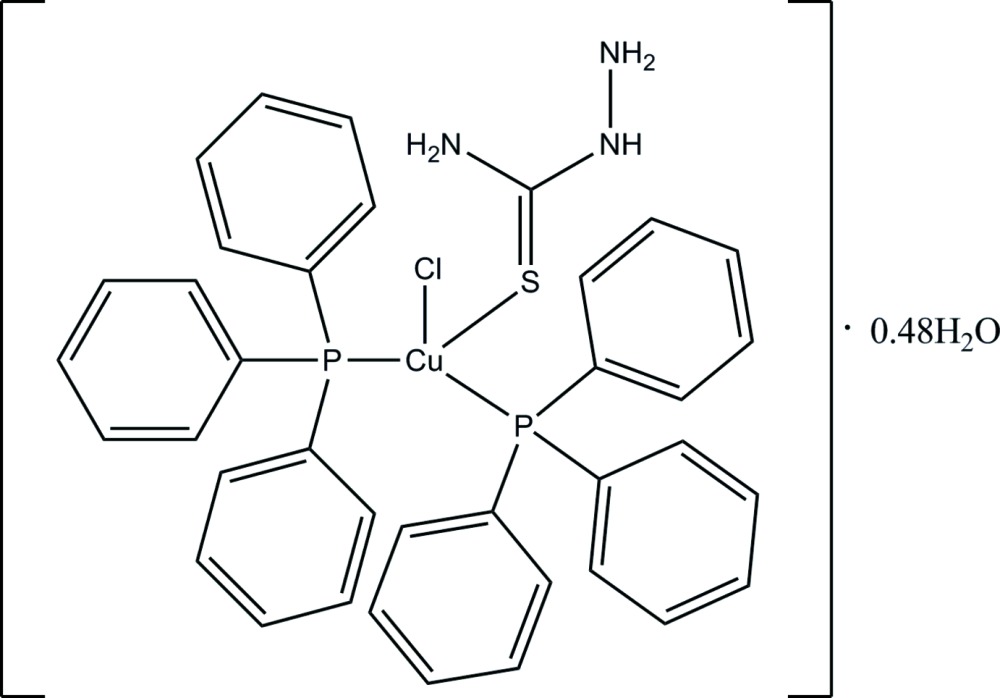


## Experimental

### 

#### Crystal data

[CuCl(CH_5_N_3_S)(C_18_H_15_P)_2_]·0.48H_2_O
*M* *_r_* = 723.29
Monoclinic, 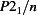

*a* = 14.8723 (7) Å
*b* = 12.4829 (6) Å
*c* = 19.2103 (9) Å
β = 96.126 (1)°
*V* = 3546.0 (3) Å^3^
*Z* = 4
Mo *K*α radiation
μ = 0.87 mm^−1^
*T* = 293 K
0.34 × 0.11 × 0.07 mm

#### Data collection

Bruker SMART APEX CCD diffractometer
Absorption correction: multi-scan (*SADABS*; Bruker, 2003) ▶ *T*_min_ = 0.880, *T*_max_ = 1
48021 measured reflections
8591 independent reflections
6691 reflections with *I* > 2σ(*I*)
*R*_int_ = 0.047

#### Refinement

*R*[*F* ^2^ > 2σ(*F* ^2^)] = 0.047
*wR*(*F* ^2^) = 0.110
*S* = 1.06
8591 reflections
428 parameters
4 restraints
H atoms treated by a mixture of independent and constrained refinement
Δρ_max_ = 0.42 e Å^−3^
Δρ_min_ = −0.19 e Å^−3^

### 

Data collection: *SMART* (Bruker, 1998 ▶); cell refinement: *SAINT* (Bruker, 2003 ▶); data reduction: *SAINT*; program(s) used to solve structure: *SHELXS97* (Sheldrick, 2008 ▶); program(s) used to refine structure: *SHELXL2012* (Sheldrick, 2008 ▶) and *SHELXLE Rev609* (Hübschle *et al.*, 2011 ▶); molecular graphics: *Mercury* (Macrae *et al.*, 2008 ▶); software used to prepare material for publication: *SHELXL97* and *publCIF* (Westrip, 2010 ▶).

## Supplementary Material

Crystal structure: contains datablock(s) I, global. DOI: 10.1107/S1600536813008556/lh5600sup1.cif

Structure factors: contains datablock(s) I. DOI: 10.1107/S1600536813008556/lh5600Isup2.hkl

Additional supplementary materials:  crystallographic information; 3D view; checkCIF report

## Figures and Tables

**Table 1:** Hydrogen-bond geometry (Å, °)

*D*—H⋯*A*	*D*—H	H⋯*A*	*D*⋯*A*	*D*—H⋯*A*
O1—H1*C*⋯Cl1	0.82 (2)	2.37 (2)	3.186 (5)	179 (12)
O1—H1*D*⋯S1^i^	0.82 (2)	2.70 (10)	3.218 (5)	123 (9)
N1—H1*B*⋯Cl1^ii^	0.86	2.48	3.302 (3)	161
N1—H1*A*⋯N3	0.86	2.27	2.628 (5)	105
N2—H2⋯Cl1	0.86	2.35	3.202 (2)	170
C42—H42⋯Cl1	0.93	2.72	3.626 (3)	164

Symmetry codes: (i) 
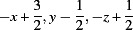
; (ii) 
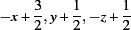
.

**Fig. 1.**
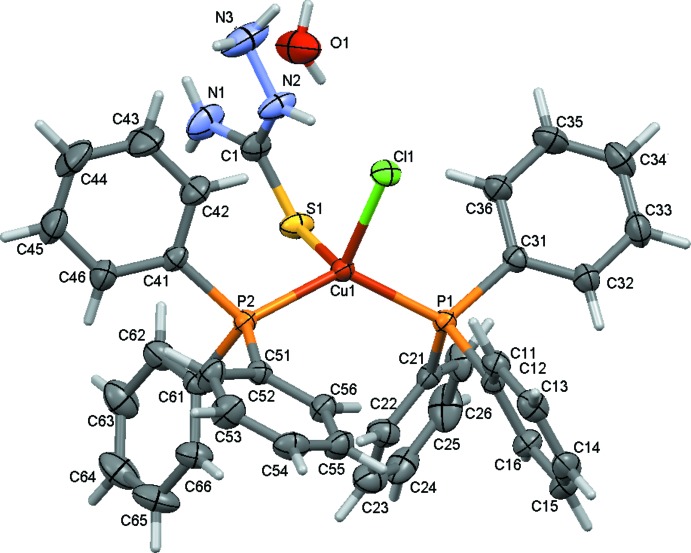
The molecular struture with displacement ellipsoids drawn at the 50% probability level.

**Fig. 2.**
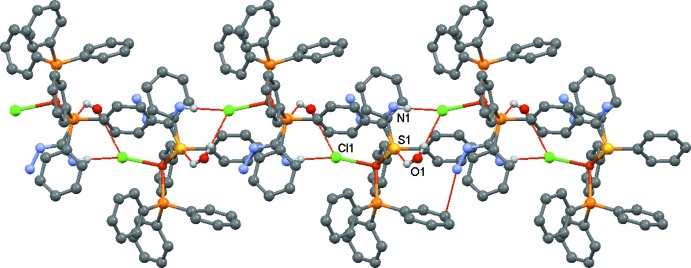
Part of the crystal structure wuth O—H···S and N—H···Cl hydrogen bonds, shown as dashed lines, which link molecules into one dimensional zigzag chain parallel to the b axis.

## supplementary crystallographic information

### Comment

Thiosemicarbazones, the condensation products of thiosemicarbazide and aldehydes, are compounds of considerable interest due to their remarkable number and variety of biological properties and the potential medical and pharmaceutical applications that result (Pelosi, 2010; Yu *et al.*, 2009). Biological applications are not limited to the thiosemicarbazones, but their metal complexes have been described to also have similar effects, and are often even more active than the uncoordinated thiosemicarbazones. Metal complexes of thiosemicarbazones have been described to be active antimicrobial agents (Alagarsamy *et al.*, 2011), are highly effective anticancer agents (Yu *et al.*, 2009), they exhibit cytotoxicity and do interact with ribonucleotide reductase (Kowol *et al.*, 2007), to name just a few of the properties they have been investigated for. The parent compound of all thiosemicarbazones, thiosemicarbazide, does also exhibit a rich metal coordination chemistry. Having no substituent at the hydrazide NH_2_ group, thiosemicarbazide is able to coordinate to metal ions in an N,*S*-chelating mode, and this is realised in most of its complexes. Of 79 complexes of thiosemicarbazide reported in the Cambridge Structural Database (2013 release, Allen, 2002), 61 featured N,*S*-chelation of the ligand, 5 displayed N,*S*-chelation and additional coordination *via* the sulfur atom, and 13 displayed mono- or bidentate coordination *via* the sulfur only. Coordination *via* only nitrogen was not observed at all. The coordination mode towards the metal dicates the conformation of the thiosemicarbazide ligand. In N,*S*-chelating complexes, the ligand necessarily features a *syn*-conformation of the sulfur atom *versus* the hydrazide NH_2_ group. In the complexes with only sulfur coordination both *syn* and anti conformation can be imagined. However, all reported examples feature the anti-conformation which is also observed in the parent compound. In salts of with protonated thiosemicarbazide cations, on the other hand, the anti conformation is again the only one realised (Andreetti *et al.*, 1970).

The reasons for the often increased biological activity of metal complexes, when compared to those of only the ligands by themselves, is manifold and depend highly on the individual case and type of biological activity under investigation. Common to many mechanisms is the need for the active compounds to pass through the cell membrane, a barrier difficult to pentrate for polar compounds such as thiosemicarbazone. Coordination to metal complexes, especially when paired with other more lipophilic ligands, reduces the polarity of the thiosemicarbazide compound. In an effort to investigate the influence of metal coordination and use of various phenyl phosphane ligands on the antibacterial properties of copper(I) and silver(I) complexes of thiosemicarbazide we reacted thiosemicarbazide with the triphenylphosphine adduct of copper(I) chloride

The use of monodenate triphenylphosphane lead to the formation or the mononuclear complex have prepared the title compound of this study, (*anti*-thiosemicabazide-*κS*)chloridobis(triphenylphosphane-*κ*P)copper(I), which crystallized from acetonitrile in the form of its hemihydrate (with a refined occupancy of 0.479 (7)). As is typical for soft metal ions such as Cu(I), thiosemicarbazide coordination is *via* the soft sulfur donor only, as had been observed for the other two Cu(I) complexes of thiosemicarbazide (Jia *et al.*, 2008*a*,*b*). Copper(II), on the other hand shows N,*S*-chelation with thiosemicarbazide (Villa *et al.*, 1972*a*,*b*; Qirong *et al.*, 1987; Chattopadhyay *et al.*,1991). In the title compound, the metal center is coordinated to one S donor of the thiosemicarbazide ligand, two P atoms of two triphenylphosphane ligands one chlorine atom. In agreement with expectation for a Cu(I) center with soft donor atoms, It displays a distorted tetrahedral coordination of the Cu^I^ center. (Fig. 1). The Cu—S bond distance of 2.3841 (7) Å is slightly longer than in the other two Cu(I) thiosemicarbazides (2.2401 (10) to 2.3192 (13) Å, Jia *et al.*, 2008*a*,*b*), but very similar to several other similar complexes such as the thiosemicarnzone complex [CuI(C_4_H_9_N_3_S)(C_18_H_15_P)_2_] with a Cu—S bond length of 2.3866 (7) Å (Wattanakanjana *et al.*, 2012). As in all other complexes with S-only coordination of thiosemicarbazides the ligand is in the *anti*-conformation, which is stablized by an intramolecular N—H···N hydrogen bond between N1—H1*A* and N3 (graph set designator as S(5) (Bernstein *et al.*, 1995)). Another intramolecular hydrogen bond, between the hydrazine N2—H2 group and the chloride anion, orients the hyrdrazide N—H group towards the chlorine atom and, through this, influences the arrangement and orientation of the ligands around the Cu^I^ center. Neighboring complexes are connected with each other through N1—H1*B*···Cl1 hydrogen bonds originating from the amide NH_2_ group, and through O1—H1*D*···S1 and O1—H1*C*···Cl1 hydrogen bonds involving the solvate water molecule. Both the direct N2—H2···Cl1 hydrogen bonds as well as the bridging H-bonds mediated by the water molecule are linking molecules with each other leading to formation of a one-dimentional zigzag chain parallel to the *b* axis (see Table and Fig.2).

### Experimental

Triphenylphosphane (0.53 g, 2 mmol) was dissolved in 30 cm^3^ of acetonitrile at 335 K. CuCl (0.10 g, 1 mmol) was added and the mixture was stirred for 1.5 h. Thiosemicarbazide (0.09 g, 1 mmol) was added and the reaction mixture was heated to reflux of the solvent for 5.5 h. The resulting clear solution was filtered and left to evaporate at room temperature. The crystalline solid, which precipitated upon standing for three days, was filtered off and dried under reduced pressure (0.38 g, yield 52%). Mp = 439–441 K. Main IR peaks (KBr, cm^-1^): *ν*(N—H) 3420, 3248, 3126 m, *ν* (C—N) + δ (N—H) 1620, 1598, 1476 m, *ν* (C=S) + *ν* (C=N) + *ν* (C—N) 1326, 1308 m, *ν* (C—N) +ν (C—S) 1082 s, *ν* (C—S) 775 s.

### Refinement

The two hydrazide NH_2_ H atoms were located in a difference Fourier map and refined isotropically, with N—H distances restrained to 0.86 (2) Å, with *U*_iso_(H) = 1.2*U*_eq_(N). The remaining H atoms bonded to N or C atoms were positioned geometrically and refined using a riding model, with C—H = 0.93, N—H = 0.86 Å with *U*_iso_(H) = 1.2*U*_eq_(C/N). A solvate water molecule is partailly occupied, the occupancy of the water molecule refined to 0.479 (7). The H atoms attached to the water molecules were located in a difference Fourier map and refined isotropically, with O—H distances restrained to 0.82 (2) Å, with *U*_iso_(H) = 1.2*U*_eq_(N).

### Crystal data

**Table:**

[CuCl(CH_5_N_3_S)(C_18_H_15_P)_2_]·0.48H_2_O	*F*(000) = 1499.2
*M**_r_* = 723.29	*D*_x_ = 1.355 Mg m^−^^3^
Monoclinic, *P*2_1_/*n*	Mo *K*α radiation, λ = 0.71073 Å
*a* = 14.8723 (7) Å	Cell parameters from 7520 reflections
*b* = 12.4829 (6) Å	θ = 2.3–23.0°
*c* = 19.2103 (9) Å	µ = 0.87 mm^−^^1^
β = 96.126 (1)°	*T* = 293 K
*V* = 3546.0 (3) Å^3^	Block, colorless
*Z* = 4	0.34 × 0.11 × 0.07 mm

### Data collection

**Table:**

Bruker SMART APEX CCD diffractometer	8591 independent reflections
Radiation source: sealed tube	6691 reflections with *I* > 2σ(*I*)
Graphite monochromator	*R*_int_ = 0.047
φ and ω scans	θ_max_ = 28.1°, θ_min_ = 1.7°
Absorption correction: multi-scan (*SADABS*; Bruker, 2003)	*h* = −19→19
*T*_min_ = 0.880, *T*_max_ = 1	*k* = −16→16
48021 measured reflections	*l* = −25→25

### Refinement

**Table:**

Refinement on *F*^2^	Primary atom site location: structure-invariant direct methods
Least-squares matrix: full	Secondary atom site location: difference Fourier map
*R*[*F*^2^ > 2σ(*F*^2^)] = 0.047	Hydrogen site location: mixed
*wR*(*F*^2^) = 0.110	H atoms treated by a mixture of independent and constrained refinement
*S* = 1.06	*w* = 1/[σ^2^(*F*_o_^2^) + (0.050*P*)^2^ + 1.0259*P*] where *P* = (*F*_o_^2^ + 2*F*_c_^2^)/3
8591 reflections	(Δ/σ)_max_ = 0.001
428 parameters	Δρ_max_ = 0.42 e Å^−^^3^
4 restraints	Δρ_min_ = −0.19 e Å^−^^3^

### Special details

**Table:**

Geometry. All e.s.d.'s (except the e.s.d. in the dihedral angle between two l.s. planes) are estimated using the full covariance matrix. The cell e.s.d.'s are taken into account individually in the estimation of e.s.d.'s in distances, angles and torsion angles; correlations between e.s.d.'s in cell parameters are only used when they are defined by crystal symmetry. An approximate (isotropic) treatment of cell e.s.d.'s is used for estimating e.s.d.'s involving l.s. planes.

### Fractional atomic coordinates and isotropic or equivalent isotropic displacement parameters (Å^2^)

**Table:**

	*x*	*y*	*z*	*U*_iso_*/*U*_eq_	Occ. (<1)
Cu1	0.87688 (2)	0.69145 (2)	0.14512 (2)	0.03859 (9)	
O1	0.6525 (4)	0.3863 (6)	0.1104 (3)	0.106 (3)	0.479 (7)
H1C	0.701 (4)	0.417 (8)	0.120 (6)	0.159*	0.479 (7)
H1D	0.631 (7)	0.336 (6)	0.130 (5)	0.159*	0.479 (7)
Cl1	0.84280 (4)	0.50261 (5)	0.14456 (3)	0.04861 (15)	
S1	0.80953 (5)	0.77828 (5)	0.23718 (4)	0.05356 (18)	
P1	1.02443 (4)	0.69509 (5)	0.19010 (3)	0.03600 (13)	
P2	0.80809 (4)	0.77112 (5)	0.04621 (3)	0.03623 (14)	
N1	0.64208 (18)	0.7719 (2)	0.27084 (16)	0.0810 (8)	
H1A	0.5922	0.7396	0.2761	0.097*	
H1B	0.6491	0.8380	0.2829	0.097*	
N2	0.69393 (15)	0.61907 (19)	0.22675 (13)	0.0648 (7)	
H2	0.7356	0.5829	0.2096	0.078*	
N3	0.6107 (2)	0.5707 (3)	0.2361 (2)	0.1044 (13)	
H3A	0.583 (3)	0.566 (4)	0.1932 (13)	0.125*	
H3B	0.618 (3)	0.5052 (19)	0.248 (3)	0.125*	
C1	0.70773 (18)	0.7203 (2)	0.24444 (13)	0.0514 (6)	
C11	1.11233 (14)	0.66548 (18)	0.13309 (11)	0.0365 (5)	
C12	1.09407 (17)	0.58379 (19)	0.08457 (13)	0.0469 (6)	
H12	1.0388	0.5483	0.0817	0.056*	
C13	1.1577 (2)	0.5551 (2)	0.04057 (14)	0.0567 (7)	
H13	1.1452	0.5004	0.0081	0.068*	
C14	1.2390 (2)	0.6069 (2)	0.04457 (15)	0.0585 (7)	
H14	1.2815	0.5874	0.0147	0.070*	
C15	1.25819 (18)	0.6875 (2)	0.09241 (16)	0.0583 (7)	
H15	1.3138	0.7222	0.0950	0.070*	
C16	1.19517 (16)	0.7173 (2)	0.13669 (13)	0.0477 (6)	
H16	1.2083	0.7722	0.1689	0.057*	
C21	1.05730 (15)	0.82700 (19)	0.22479 (13)	0.0418 (5)	
C22	1.0509 (2)	0.9125 (2)	0.17833 (15)	0.0582 (7)	
H22	1.0288	0.9006	0.1318	0.070*	
C23	1.0763 (2)	1.0143 (2)	0.1993 (2)	0.0718 (9)	
H23	1.0726	1.0702	0.1671	0.086*	
C24	1.1072 (2)	1.0327 (3)	0.2678 (2)	0.0782 (10)	
H24	1.1258	1.1011	0.2822	0.094*	
C25	1.1109 (2)	0.9510 (3)	0.3150 (2)	0.0877 (12)	
H25	1.1304	0.9644	0.3618	0.105*	
C26	1.0856 (2)	0.8474 (3)	0.29384 (16)	0.0646 (8)	
H26	1.0879	0.7923	0.3265	0.077*	
C31	1.05437 (16)	0.60214 (19)	0.26296 (12)	0.0419 (5)	
C32	1.14238 (18)	0.5696 (2)	0.28240 (14)	0.0559 (7)	
H32	1.1889	0.5962	0.2586	0.067*	
C33	1.1621 (2)	0.4978 (3)	0.33684 (16)	0.0677 (8)	
H33	1.2217	0.4774	0.3498	0.081*	
C34	1.0941 (2)	0.4567 (3)	0.37161 (16)	0.0727 (9)	
H34	1.1074	0.4085	0.4082	0.087*	
C35	1.0072 (2)	0.4869 (3)	0.35232 (16)	0.0747 (9)	
H35	0.9611	0.4581	0.3755	0.090*	
C36	0.98627 (19)	0.5600 (2)	0.29870 (14)	0.0573 (7)	
H36	0.9266	0.5807	0.2867	0.069*	
C41	0.68669 (15)	0.7430 (2)	0.02893 (12)	0.0422 (5)	
C42	0.65597 (19)	0.6448 (3)	0.04902 (16)	0.0626 (7)	
H42	0.6965	0.5956	0.0710	0.075*	
C43	0.5651 (2)	0.6188 (3)	0.0366 (2)	0.0890 (11)	
H43	0.5449	0.5524	0.0503	0.107*	
C44	0.5049 (2)	0.6905 (4)	0.0044 (2)	0.0912 (12)	
H44	0.4439	0.6731	−0.0035	0.109*	
C45	0.5344 (2)	0.7876 (3)	−0.01622 (19)	0.0780 (10)	
H45	0.4935	0.8360	−0.0386	0.094*	
C46	0.62496 (18)	0.8145 (3)	−0.00402 (16)	0.0610 (7)	
H46	0.6445	0.8812	−0.0180	0.073*	
C51	0.85129 (15)	0.73927 (18)	−0.03696 (12)	0.0389 (5)	
C52	0.79750 (19)	0.7307 (3)	−0.10006 (14)	0.0580 (7)	
H52	0.7360	0.7458	−0.1022	0.070*	
C53	0.8345 (2)	0.6999 (3)	−0.16006 (15)	0.0660 (8)	
H53	0.7975	0.6944	−0.2021	0.079*	
C54	0.9242 (2)	0.6776 (2)	−0.15837 (15)	0.0587 (7)	
H54	0.9482	0.6560	−0.1989	0.070*	
C55	0.97882 (19)	0.6872 (2)	−0.09687 (15)	0.0576 (7)	
H55	1.0404	0.6735	−0.0956	0.069*	
C56	0.94248 (17)	0.7172 (2)	−0.03655 (14)	0.0486 (6)	
H56	0.9801	0.7226	0.0052	0.058*	
C61	0.80914 (16)	0.91750 (19)	0.04859 (12)	0.0429 (5)	
C62	0.7576 (2)	0.9697 (2)	0.09473 (15)	0.0605 (7)	
H62	0.7245	0.9294	0.1238	0.073*	
C63	0.7549 (3)	1.0802 (2)	0.09799 (17)	0.0766 (9)	
H63	0.7194	1.1138	0.1286	0.092*	
C64	0.8040 (3)	1.1398 (3)	0.05644 (19)	0.0857 (11)	
H64	0.8020	1.2142	0.0584	0.103*	
C65	0.8562 (3)	1.0901 (3)	0.01195 (19)	0.0883 (11)	
H65	0.8905	1.1310	−0.0158	0.106*	
C66	0.8586 (2)	0.9793 (2)	0.00755 (15)	0.0623 (7)	
H66	0.8941	0.9467	−0.0234	0.075*	

### Atomic displacement parameters (Å^2^)

**Table:**

	*U*^11^	*U*^22^	*U*^33^	*U*^12^	*U*^13^	*U*^23^
Cu1	0.03296 (15)	0.04514 (17)	0.03791 (16)	−0.00235 (12)	0.00491 (11)	0.00367 (12)
O1	0.104 (5)	0.132 (6)	0.074 (4)	−0.054 (4)	−0.025 (3)	0.031 (3)
Cl1	0.0553 (4)	0.0380 (3)	0.0546 (4)	−0.0016 (3)	0.0157 (3)	−0.0045 (3)
S1	0.0600 (4)	0.0470 (4)	0.0581 (4)	−0.0062 (3)	0.0267 (3)	−0.0090 (3)
P1	0.0326 (3)	0.0408 (3)	0.0345 (3)	−0.0015 (2)	0.0031 (2)	0.0009 (2)
P2	0.0323 (3)	0.0405 (3)	0.0359 (3)	−0.0013 (2)	0.0036 (2)	0.0022 (2)
N1	0.0616 (16)	0.0768 (18)	0.111 (2)	0.0057 (14)	0.0397 (16)	−0.0275 (16)
N2	0.0507 (13)	0.0628 (15)	0.0868 (17)	−0.0123 (11)	0.0347 (12)	−0.0253 (13)
N3	0.068 (2)	0.099 (2)	0.156 (3)	−0.0340 (19)	0.055 (2)	−0.046 (3)
C1	0.0513 (15)	0.0576 (16)	0.0480 (14)	0.0054 (12)	0.0178 (12)	−0.0065 (12)
C11	0.0350 (11)	0.0408 (12)	0.0339 (11)	0.0038 (9)	0.0047 (9)	0.0038 (9)
C12	0.0510 (14)	0.0423 (13)	0.0475 (14)	−0.0025 (11)	0.0055 (11)	−0.0006 (11)
C13	0.0751 (19)	0.0470 (15)	0.0499 (15)	0.0075 (14)	0.0155 (13)	−0.0055 (12)
C14	0.0648 (18)	0.0579 (17)	0.0574 (16)	0.0172 (14)	0.0276 (14)	0.0085 (13)
C15	0.0424 (14)	0.0652 (18)	0.0700 (18)	−0.0009 (12)	0.0180 (13)	0.0062 (15)
C16	0.0407 (13)	0.0538 (15)	0.0491 (14)	−0.0042 (11)	0.0061 (11)	−0.0041 (11)
C21	0.0315 (11)	0.0457 (13)	0.0484 (13)	−0.0006 (9)	0.0057 (10)	−0.0063 (10)
C22	0.0693 (18)	0.0483 (15)	0.0594 (17)	−0.0014 (13)	0.0173 (14)	−0.0006 (13)
C23	0.074 (2)	0.0436 (16)	0.102 (3)	−0.0007 (14)	0.0286 (19)	−0.0052 (16)
C24	0.0532 (18)	0.0538 (18)	0.126 (3)	−0.0037 (14)	0.0035 (19)	−0.031 (2)
C25	0.086 (2)	0.080 (2)	0.088 (3)	0.009 (2)	−0.030 (2)	−0.039 (2)
C26	0.0679 (19)	0.0608 (17)	0.0601 (17)	0.0080 (15)	−0.0159 (14)	−0.0101 (14)
C31	0.0436 (13)	0.0444 (13)	0.0368 (12)	0.0000 (10)	0.0006 (10)	0.0007 (10)
C32	0.0508 (15)	0.0623 (17)	0.0540 (15)	0.0066 (13)	0.0028 (12)	0.0120 (13)
C33	0.0650 (19)	0.072 (2)	0.0642 (18)	0.0212 (16)	−0.0008 (15)	0.0146 (15)
C34	0.096 (3)	0.0641 (19)	0.0569 (18)	0.0155 (18)	0.0017 (17)	0.0196 (15)
C35	0.081 (2)	0.085 (2)	0.0607 (19)	−0.0052 (18)	0.0165 (16)	0.0279 (17)
C36	0.0543 (16)	0.0706 (18)	0.0470 (15)	−0.0003 (14)	0.0062 (12)	0.0146 (13)
C41	0.0337 (11)	0.0549 (15)	0.0376 (12)	−0.0034 (10)	0.0021 (9)	−0.0042 (10)
C42	0.0473 (15)	0.0670 (18)	0.0710 (19)	−0.0138 (13)	−0.0052 (13)	0.0002 (15)
C43	0.062 (2)	0.096 (3)	0.106 (3)	−0.037 (2)	−0.0070 (19)	0.006 (2)
C44	0.0389 (17)	0.141 (4)	0.092 (3)	−0.019 (2)	−0.0038 (16)	−0.006 (2)
C45	0.0416 (16)	0.110 (3)	0.080 (2)	0.0091 (17)	−0.0061 (15)	0.003 (2)
C46	0.0427 (15)	0.0740 (19)	0.0654 (18)	0.0024 (13)	0.0007 (13)	0.0113 (15)
C51	0.0401 (12)	0.0391 (12)	0.0381 (12)	−0.0010 (10)	0.0075 (9)	0.0010 (9)
C52	0.0441 (14)	0.086 (2)	0.0431 (14)	0.0032 (14)	0.0008 (11)	−0.0040 (14)
C53	0.0654 (19)	0.093 (2)	0.0395 (14)	−0.0050 (16)	0.0033 (13)	−0.0071 (14)
C54	0.0719 (19)	0.0564 (17)	0.0520 (16)	−0.0060 (14)	0.0265 (14)	−0.0076 (12)
C55	0.0478 (15)	0.0610 (17)	0.0672 (18)	0.0020 (13)	0.0206 (13)	−0.0021 (14)
C56	0.0417 (13)	0.0567 (16)	0.0477 (14)	0.0002 (11)	0.0063 (11)	−0.0009 (11)
C61	0.0479 (13)	0.0395 (12)	0.0401 (12)	−0.0013 (10)	−0.0011 (10)	0.0033 (10)
C62	0.077 (2)	0.0506 (16)	0.0557 (16)	0.0073 (14)	0.0170 (14)	0.0043 (13)
C63	0.111 (3)	0.0526 (18)	0.067 (2)	0.0171 (18)	0.0143 (19)	−0.0046 (15)
C64	0.140 (3)	0.0417 (17)	0.073 (2)	−0.002 (2)	0.003 (2)	−0.0008 (16)
C65	0.136 (3)	0.0539 (19)	0.079 (2)	−0.027 (2)	0.030 (2)	0.0082 (17)
C66	0.079 (2)	0.0498 (16)	0.0603 (17)	−0.0128 (14)	0.0192 (15)	0.0005 (13)

### Geometric parameters (Å, º)

**Table:**

Cu1—P1	2.2714 (6)	C31—C36	1.387 (3)
Cu1—P2	2.2872 (6)	C32—C33	1.385 (4)
Cu1—S1	2.3841 (7)	C32—H32	0.9300
Cu1—Cl1	2.4110 (7)	C33—C34	1.369 (5)
O1—H1C	0.82 (2)	C33—H33	0.9300
O1—H1D	0.82 (2)	C34—C35	1.359 (5)
S1—C1	1.697 (3)	C34—H34	0.9300
P1—C21	1.823 (2)	C35—C36	1.386 (4)
P1—C11	1.831 (2)	C35—H35	0.9300
P1—C31	1.836 (2)	C36—H36	0.9300
P2—C61	1.828 (2)	C41—C42	1.377 (4)
P2—C51	1.828 (2)	C41—C46	1.385 (4)
P2—C41	1.834 (2)	C42—C43	1.386 (4)
N1—C1	1.316 (3)	C42—H42	0.9300
N1—H1A	0.8600	C43—C44	1.366 (5)
N1—H1B	0.8600	C43—H43	0.9300
N2—C1	1.319 (3)	C44—C45	1.362 (5)
N2—N3	1.406 (4)	C44—H44	0.9300
N2—H2	0.8600	C45—C46	1.383 (4)
N3—H3A	0.879 (19)	C45—H45	0.9300
N3—H3B	0.851 (19)	C46—H46	0.9300
C11—C16	1.387 (3)	C51—C56	1.383 (3)
C11—C12	1.389 (3)	C51—C52	1.383 (3)
C12—C13	1.382 (4)	C52—C53	1.384 (4)
C12—H12	0.9300	C52—H52	0.9300
C13—C14	1.365 (4)	C53—C54	1.359 (4)
C13—H13	0.9300	C53—H53	0.9300
C14—C15	1.373 (4)	C54—C55	1.366 (4)
C14—H14	0.9300	C54—H54	0.9300
C15—C16	1.382 (4)	C55—C56	1.382 (4)
C15—H15	0.9300	C55—H55	0.9300
C16—H16	0.9300	C56—H56	0.9300
C21—C26	1.372 (4)	C61—C66	1.372 (4)
C21—C22	1.388 (4)	C61—C62	1.394 (4)
C22—C23	1.373 (4)	C62—C63	1.382 (4)
C22—H22	0.9300	C62—H62	0.9300
C23—C24	1.367 (5)	C63—C64	1.360 (5)
C23—H23	0.9300	C63—H63	0.9300
C24—C25	1.362 (5)	C64—C65	1.364 (5)
C24—H24	0.9300	C64—H64	0.9300
C25—C26	1.395 (5)	C65—C66	1.386 (4)
C25—H25	0.9300	C65—H65	0.9300
C26—H26	0.9300	C66—H66	0.9300
C31—C32	1.383 (3)		
			
P1—Cu1—P2	129.47 (2)	C36—C31—P1	119.14 (18)
P1—Cu1—S1	100.02 (3)	C31—C32—C33	120.8 (3)
P2—Cu1—S1	103.44 (3)	C31—C32—H32	119.6
P1—Cu1—Cl1	102.44 (2)	C33—C32—H32	119.6
P2—Cu1—Cl1	110.26 (2)	C34—C33—C32	120.2 (3)
S1—Cu1—Cl1	110.06 (2)	C34—C33—H33	119.9
H1C—O1—H1D	129 (10)	C32—C33—H33	119.9
C1—S1—Cu1	108.44 (9)	C35—C34—C33	119.6 (3)
C21—P1—C11	102.72 (10)	C35—C34—H34	120.2
C21—P1—C31	104.98 (11)	C33—C34—H34	120.2
C11—P1—C31	101.42 (10)	C34—C35—C36	121.1 (3)
C21—P1—Cu1	111.56 (8)	C34—C35—H35	119.4
C11—P1—Cu1	119.40 (7)	C36—C35—H35	119.4
C31—P1—Cu1	115.04 (8)	C35—C36—C31	120.0 (3)
C61—P2—C51	103.67 (11)	C35—C36—H36	120.0
C61—P2—C41	101.63 (11)	C31—C36—H36	120.0
C51—P2—C41	103.36 (10)	C42—C41—C46	118.6 (2)
C61—P2—Cu1	114.33 (8)	C42—C41—P2	118.0 (2)
C51—P2—Cu1	117.63 (8)	C46—C41—P2	123.4 (2)
C41—P2—Cu1	114.30 (8)	C41—C42—C43	120.4 (3)
C1—N1—H1A	120.0	C41—C42—H42	119.8
C1—N1—H1B	120.0	C43—C42—H42	119.8
H1A—N1—H1B	120.0	C44—C43—C42	120.3 (3)
C1—N2—N3	119.6 (2)	C44—C43—H43	119.9
C1—N2—H2	120.2	C42—C43—H43	119.9
N3—N2—H2	120.2	C45—C44—C43	119.9 (3)
N2—N3—H3A	104 (3)	C45—C44—H44	120.0
N2—N3—H3B	111 (3)	C43—C44—H44	120.0
H3A—N3—H3B	102 (4)	C44—C45—C46	120.3 (3)
N1—C1—N2	117.8 (3)	C44—C45—H45	119.8
N1—C1—S1	121.9 (2)	C46—C45—H45	119.8
N2—C1—S1	120.19 (19)	C45—C46—C41	120.4 (3)
C16—C11—C12	119.0 (2)	C45—C46—H46	119.8
C16—C11—P1	124.28 (18)	C41—C46—H46	119.8
C12—C11—P1	116.67 (17)	C56—C51—C52	117.7 (2)
C13—C12—C11	120.2 (2)	C56—C51—P2	118.30 (18)
C13—C12—H12	119.9	C52—C51—P2	123.93 (19)
C11—C12—H12	119.9	C51—C52—C53	120.5 (3)
C14—C13—C12	120.2 (3)	C51—C52—H52	119.8
C14—C13—H13	119.9	C53—C52—H52	119.8
C12—C13—H13	119.9	C54—C53—C52	120.9 (3)
C13—C14—C15	120.3 (2)	C54—C53—H53	119.6
C13—C14—H14	119.8	C52—C53—H53	119.6
C15—C14—H14	119.8	C53—C54—C55	119.6 (3)
C14—C15—C16	120.2 (3)	C53—C54—H54	120.2
C14—C15—H15	119.9	C55—C54—H54	120.2
C16—C15—H15	119.9	C54—C55—C56	119.9 (3)
C15—C16—C11	120.1 (2)	C54—C55—H55	120.0
C15—C16—H16	120.0	C56—C55—H55	120.0
C11—C16—H16	120.0	C55—C56—C51	121.4 (2)
C26—C21—C22	118.1 (2)	C55—C56—H56	119.3
C26—C21—P1	124.3 (2)	C51—C56—H56	119.3
C22—C21—P1	117.53 (19)	C66—C61—C62	117.9 (2)
C23—C22—C21	121.6 (3)	C66—C61—P2	123.5 (2)
C23—C22—H22	119.2	C62—C61—P2	118.63 (19)
C21—C22—H22	119.2	C63—C62—C61	121.1 (3)
C24—C23—C22	119.5 (3)	C63—C62—H62	119.5
C24—C23—H23	120.2	C61—C62—H62	119.5
C22—C23—H23	120.2	C64—C63—C62	120.0 (3)
C25—C24—C23	120.0 (3)	C64—C63—H63	120.0
C25—C24—H24	120.0	C62—C63—H63	120.0
C23—C24—H24	120.0	C63—C64—C65	119.7 (3)
C24—C25—C26	120.7 (3)	C63—C64—H64	120.1
C24—C25—H25	119.7	C65—C64—H64	120.1
C26—C25—H25	119.7	C64—C65—C66	120.9 (3)
C21—C26—C25	119.9 (3)	C64—C65—H65	119.6
C21—C26—H26	120.0	C66—C65—H65	119.6
C25—C26—H26	120.0	C61—C66—C65	120.4 (3)
C32—C31—C36	118.3 (2)	C61—C66—H66	119.8
C32—C31—P1	122.49 (19)	C65—C66—H66	119.8
			
N3—N2—C1—N1	0.5 (5)	C32—C31—C36—C35	−0.1 (4)
N3—N2—C1—S1	177.1 (3)	P1—C31—C36—C35	177.8 (2)
Cu1—S1—C1—N1	−154.9 (2)	C61—P2—C41—C42	−156.0 (2)
Cu1—S1—C1—N2	28.6 (3)	C51—P2—C41—C42	96.8 (2)
C21—P1—C11—C16	18.2 (2)	Cu1—P2—C41—C42	−32.3 (2)
C31—P1—C11—C16	−90.2 (2)	C61—P2—C41—C46	25.0 (3)
Cu1—P1—C11—C16	142.20 (18)	C51—P2—C41—C46	−82.3 (2)
C21—P1—C11—C12	−163.36 (18)	Cu1—P2—C41—C46	148.6 (2)
C31—P1—C11—C12	88.22 (19)	C46—C41—C42—C43	−0.3 (4)
Cu1—P1—C11—C12	−39.3 (2)	P2—C41—C42—C43	−179.4 (3)
C16—C11—C12—C13	−0.1 (4)	C41—C42—C43—C44	0.0 (6)
P1—C11—C12—C13	−178.7 (2)	C42—C43—C44—C45	0.5 (6)
C11—C12—C13—C14	0.0 (4)	C43—C44—C45—C46	−0.6 (6)
C12—C13—C14—C15	0.2 (4)	C44—C45—C46—C41	0.3 (5)
C13—C14—C15—C16	−0.3 (4)	C42—C41—C46—C45	0.1 (4)
C14—C15—C16—C11	0.2 (4)	P2—C41—C46—C45	179.2 (2)
C12—C11—C16—C15	0.0 (4)	C61—P2—C51—C56	95.5 (2)
P1—C11—C16—C15	178.4 (2)	C41—P2—C51—C56	−158.8 (2)
C11—P1—C21—C26	−114.0 (2)	Cu1—P2—C51—C56	−31.8 (2)
C31—P1—C21—C26	−8.3 (3)	C61—P2—C51—C52	−87.8 (2)
Cu1—P1—C21—C26	117.0 (2)	C41—P2—C51—C52	17.9 (3)
C11—P1—C21—C22	67.8 (2)	Cu1—P2—C51—C52	144.9 (2)
C31—P1—C21—C22	173.51 (19)	C56—C51—C52—C53	0.7 (4)
Cu1—P1—C21—C22	−61.3 (2)	P2—C51—C52—C53	−176.0 (2)
C26—C21—C22—C23	3.4 (4)	C51—C52—C53—C54	−0.1 (5)
P1—C21—C22—C23	−178.3 (2)	C52—C53—C54—C55	−0.9 (5)
C21—C22—C23—C24	−1.3 (5)	C53—C54—C55—C56	1.4 (4)
C22—C23—C24—C25	−1.3 (5)	C54—C55—C56—C51	−0.8 (4)
C23—C24—C25—C26	1.7 (5)	C52—C51—C56—C55	−0.3 (4)
C22—C21—C26—C25	−2.9 (4)	P2—C51—C56—C55	176.6 (2)
P1—C21—C26—C25	178.8 (2)	C51—P2—C61—C66	−18.1 (3)
C24—C25—C26—C21	0.5 (5)	C41—P2—C61—C66	−125.2 (2)
C21—P1—C31—C32	−77.2 (2)	Cu1—P2—C61—C66	111.2 (2)
C11—P1—C31—C32	29.5 (2)	C51—P2—C61—C62	162.3 (2)
Cu1—P1—C31—C32	159.82 (19)	C41—P2—C61—C62	55.3 (2)
C21—P1—C31—C36	104.9 (2)	Cu1—P2—C61—C62	−68.4 (2)
C11—P1—C31—C36	−148.4 (2)	C66—C61—C62—C63	1.4 (4)
Cu1—P1—C31—C36	−18.1 (2)	P2—C61—C62—C63	−179.1 (2)
C36—C31—C32—C33	−0.9 (4)	C61—C62—C63—C64	−0.9 (5)
P1—C31—C32—C33	−178.8 (2)	C62—C63—C64—C65	−0.3 (6)
C31—C32—C33—C34	1.0 (5)	C63—C64—C65—C66	1.2 (6)
C32—C33—C34—C35	0.0 (5)	C62—C61—C66—C65	−0.6 (4)
C33—C34—C35—C36	−1.1 (5)	P2—C61—C66—C65	179.9 (3)
C34—C35—C36—C31	1.1 (5)	C64—C65—C66—C61	−0.7 (6)

### Hydrogen-bond geometry (Å, º)

**Table:**

*D*—H···*A*	*D*—H	H···*A*	*D*···*A*	*D*—H···*A*
O1—H1*C*···Cl1	0.82 (2)	2.37 (2)	3.186 (5)	179 (12)
O1—H1*D*···S1^i^	0.82 (2)	2.70 (10)	3.218 (5)	123 (9)
N1—H1*B*···Cl1^ii^	0.86	2.48	3.302 (3)	161
N1—H1*A*···N3	0.86	2.27	2.628 (5)	105
N2—H2···Cl1	0.86	2.35	3.202 (2)	170
C42—H42···Cl1	0.93	2.72	3.626 (3)	164

Symmetry codes: (i) −*x*+3/2, *y*−1/2, −*z*+1/2; (ii) −*x*+3/2, *y*+1/2, −*z*+1/2.
